# Morphological and Transcriptome Analyses Provide Insights into Growth Inhibition of *Trichophyton rubrum* Caused by Laser Irradiation

**DOI:** 10.1155/2020/6052461

**Published:** 2020-04-16

**Authors:** Rui-Na Zhang, Jun-Ying Zhao, Lin-Feng Li

**Affiliations:** Department of Dermatology, Beijing Friendship Hospital, Capital Medical University, Beijing 100050, China

## Abstract

*Trichophyton rubrum* is one of the most common types of dermatophyte, causing superficial skin mycosis in human populations. Although laser treatment of onychomycosis has been proven to be effective in the clinic, the underlying mechanism of the effect of the laser on fungal growth is not clear. The objective of the present study was to observe the ultrastructural changes of *Trichophyton rubrum* following laser irradiation and compare the transcriptome differences between the laser irradiation group and control group. In the present study, scanning electron microscopy and transmission electron microscopy were used to observe the ultrastructural changes following the laser irradiation of *Trichophyton rubrum.* We also performed RNA-seq to investigate the effects of laser irradiation on *Trichophyton rubrum* by comparing the transcriptome pattern with the control. Morphological observation with electron microscopy indicated that laser irradiation resulted in the destruction of the cell membrane system. A significant induction of apoptosis was noted compared with the control group, which was confirmed by the formation of the myeloid body and protein aggregates in the cytoplasm. RNA-seq demonstrated that the expression levels of Acyl-CoA N-acyltransferase and S-adenosyl-L-methionine-dependent methyltransferase were increased in the laser irradiation group. This result indicated that laser irradiation triggered the initiation of the damage repair pathway. In conclusion, the present study suggested that laser irradiation caused physiological injury and therefore inhibited the growth of *Trichophyton rubrum.*

## 1. Introduction

Dermatophytes are highly specialized pathogenic fungi, which invade the skin, nails, and hair of humans and the corresponding parts of animals, including hides, hooves, feathers, beaks, and other dermal appendages [1]. *Trichophyton rubrum* (*T. rubrum*) is one of the most common types of dermatophytes, causing superficial skin mycosis in human populations [2, 3]. The application of several types of lasers in the treatment of mycosis and onychomycosis has been employed recently as an effective treatment in clinical practice [4–6]. The mechanism of laser treatment on fungal nail infection is not well known, but there are several hypotheses. One hypothesis is that laser energy may be preferentially absorbed by fungal pathogens since the existence of differences in thermal conductivity between fungi and surrounding human tissues [7]. Another hypothesis is that high concentration of pigment deposition in *T. rubrum* results in free radicals by absorbing intensive laser energy [8].

**Figure 1:**
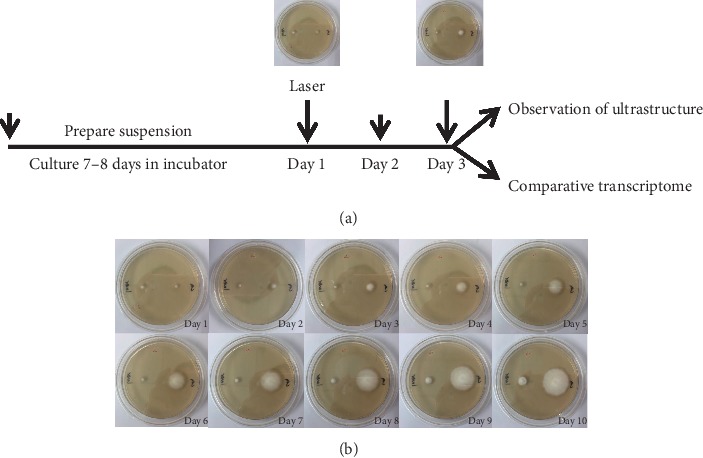
Design of the experimental procedure and growth inhibition of *T. rubrum* induced by laser irradiation. (a) Schematic representation of the experimental procedure. The suspension was prepared and inoculated in SDA for 7-8 days. When the diameter of the colony reached 6 mm, laser irradiation was performed on the laser side (day 1). The ultrastructure was observed, and the transcriptome was compared on the third day following laser exposure. (b) The growth of colonies to a 6 mm diameter length was inhibited for 7 days following single laser irradiation. The left side of each medium is the laser group, and the right side is the control group.

However, additional *in vitro* experiments derived from different studies have produced different results [9–11]. Vural et al. [9] and Ghavam et al. [10] observed that both the 1064 nm Q-switched Nd:YAG laser and the 532 nm Q-switched Nd:YAG laser could inhibit the growth of *T. rubrum* in an *in vitro* study. Nevertheless, Kim et al. [11] irradiated five clinical strains of *T. rubrum* with a long-pulse width 1064 nm Nd:YAG laser and observed no significant effect on growth. Clinical trials have shown that laser therapy is effective for the treatment of onychomycosis, but the results of *in vitro* studies are conflicting. So in the early stage of our study, various *in vitro* experimental conditions were tested to identify the condition that can inhibit the growth of *T. rubrum* following the irradiation of the colony (Zhang, Zhao and Li, unpublished data). We considered the influence of several factors, such as colony size, laser energy density, number of irradiation, and number of irradiation spots in order to identify the conditions that efficiently inhibit the growth of colonies following laser irradiation. Under these specific conditions, the growth of *T. rubrum* was inhibited for 7 days following laser irradiation (as shown in Figure 1). Subsequently, additional experiments were conducted in order to explore the morphological changes of long pulse Nd: YAG 1064 nm laser irradiation on the growth of *T. rubrum* and assess the differences in the transcriptome. The morphological observations by electron microscopy indicated that laser irradiation resulted in the destruction of the cell membrane. In addition, the induction of apoptosis was evident by the formation of the myeloid body and protein aggregates in the cytoplasm. The results of the RNA-seq analysis indicated that the laser irradiation triggered the initiation of the damage repair pathway. Taken collectively, the data indicated that the laser irradiation caused physical injury and therefore inhibited the growth of *T. rubrum*.

## 2. Materials and Methods

### 2.1. Suspension Preparation and Laser Irradiation

Schematic representation of the experimental procedure is shown in Figure 1(a).

A treatment sensitive strain of *T. rubrum* was inoculated on Sabouraud dextrose agar (SDA, Beijing Ginko-forest science Co., Ltd) at 25°C for activation and purification for 7-8 days. Sterile saline solution (0.85%, 1 ml) was added into the culture dish, gently aspirated with a Pasteur pipette, transferred to a sterile test tube, and left for 3–5 min. The upper liquid (including spores and hyphae) was removed, shaken for 15 s, and placed in a turbidimetric tube to determine its concentration using a turbidimeter. The final turbidity was adjusted to 1.0 mcf. A total of 1.0 *μ*l suspension was inoculated on the left and right sides of the SDA. One side was used for the control group and the other for the laser group. The dish was cultured at 25°C, and the growth of the colony was observed every day. When the colony reached 6 mm in diameter, laser irradiation was performed on the colony of the laser side (on the left side in Figure 1(b)). The equipment and parameter settings used were as follows: long pulse Nd:YAG 1064 nm laser (Beijing Shiji Guangtong Biotechnology Co., Ltd.), 3 mm spot size, 1 Hz frequency, 30 ms pulse, and 408 J/cm^2^. The 6 mm colony was composed of 200 spots. On the third day following laser exposure, the morphology was observed by electron microscopy and the transcriptome was used to compare the differences between the laser-irradiated and the control groups.

### 2.2. Morphological Observation of *T. rubrum* following Laser Irradiation

A single colony from the laser and the control groups was selected, respectively, for SEM analysis following 3 days of culture following irradiation. The mycelia were washed thrice in phosphate buffer (pH 7.0) and subsequently fixed with 3% glutaraldehyde for 2 h at 4°C. Subsequently, the specimens were soaked in 1% osmic acid for 2 h at 4°C. Each specimen was subjected to dehydration by ethanol at the following concentrations: 30%, 50%, 60%, 70%, 80%, 90%, 95%, and 100% sequentially, for 20 min each time. The samples were dehydrated with acetone for three times, for 20 min each time. The samples were subsequently mounted on stubs over carbon tape, coated with gold-palladium powder using a sputter coater and finally observed by scanning electron microscopy (TM-1000, HITACHI, Japan).

The fungal mycelia were prepared for TEM analysis by fixation and dehydration with a method similar to that reported for SEM. Following dehydration, the specimens were embedded in Epon812 epoxy resin, sectioned to ultrathin slices, and stained with uranium acetate and lead citrate, sequentially. Subsequently, the specimens were observed by TEM (TM-1000, HITACHI, Japan).

### 2.3. RNA Sequencing and Data Analysis

Two groups (3 samples each) of *T. rubrum* were cultured in a 100 ml flask, which was placed in a 28°C culture shaker at a speed of 150 r/min for 5 days. One group was treated with laser irradiation, and the other was kept as the control group. Subsequently, the fungal samples were centrifuged, and the supernatant was discarded. The pellet was collected, frozen in liquid nitrogen, and preserved at −80°C prior to RNA extraction.

Total RNA was extracted by the TRIzol reagent (Invitrogen, USA). The cDNA libraries from each sample were prepared with the NEBNext® Ultra™ RNA Library Prep Kit for Illumina (NEB, USA). Sequencing was performed on an Illumina HiSeq 2500 instrument at Novogene Bioinformatics Institute, Beijing, China.

We trimmed reads by removing adapter sequences, reads with excess number (5%) of unknown base calls (*N*), and low-quality bases using Trimmomatic v0.36 [12] with the following parameter settings—LEADING : 3, TRAILING : 3, SLIDINGWINDOW : 4 : 15, and MINLEN : 40. The reference genome and the gene model annotation files were downloaded from NCBI (*Trichophyton rubrum* CBS 118892, https://www.ncbi.nlm.nih.gov/genome/799?genome_assembly_id=30853). The index of the reference genome was built using Hisat2 v2.0.5 [13], and the pair-end clean reads were aligned to the reference genome using Hisat2 v2.0.5 with default parameters. We selected Hisat2 as the mapping tool since this software could generate a database of splice junctions based on the gene model annotation file. Therefore, a better mapping result than the other nonsplice mapping tools could be obtained.

### 2.4. Data Processing and Statistical Analysis

Feature Counts v1.5.0-p3 [14] was used to count the read numbers mapped to each gene. The gene expression levels were measured using the criteria of reads per kb per million mapped reads (RPKM). Genes differentially expressed in the laser-treated and control groups were identified as follows: nonnormalized read counts for all detected genes were acquired by StringTie v1.3.3b [15] and a reads count table was generated by the Python script “prepDE.py” in the StringTie package. Then, the differentially expressed genes (DEGs) were identified by negative binomial generalized linear models implemented in DESeq2 software v1.20.0 [16]. In total, 346 DEGs were identified with a |log_2_ (fold change)| > 2 and adjusted *p* value < 0.05 (using the Benjamini–Hochberg algorithm).

To examine the differences in gene expression between the laser-treated and the control groups, we also performed a hierarchical clustering of gene expression level profile on the log (RPKM) using the heatmap.2 function in the R package gplots v3.0.1.1 [17]. Furthermore, GO enrichment analysis was performed by the GOseq R package v1.24.0 [18], and the gene length bias was corrected. KEGG enrichment was performed by the KOBAS software v3.0.3 [19]. Significance analysis was performed by Fisher's exact test.

## 3. Results

### 3.1. Growth of *T. rubrum* Was Inhibited by Laser Irradiation

The growth of *T. rubrum* was substantially inhibited in the laser irradiation group. The colony size of the fungi that was irradiated by the long pulse Nd:YAG 1064 nm laser was smaller than that of the untreated control. However, the colony of laser group gradually restored its growth ability with time (Figure 1(b)).

### 3.2. Ultrastructural Changes of *T. rubrum* following Laser Irradiation

**Figure 2:**
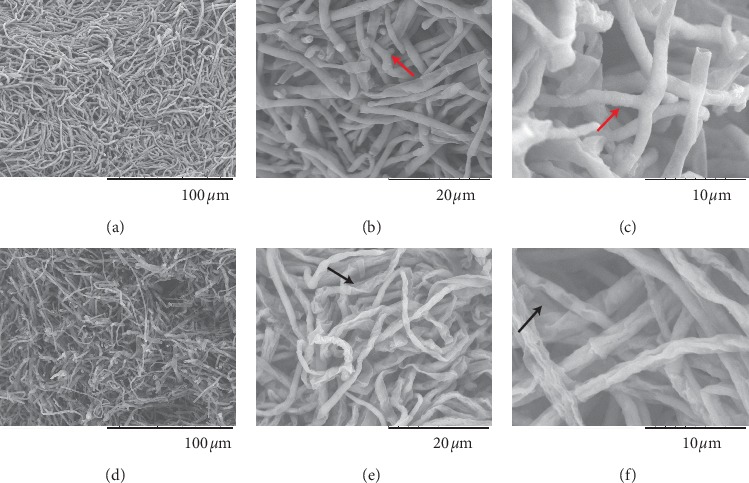
SEM observation of *T. rubrum* in the control group and laser groups. (a) (SEM × 1000) Large amount of hyphae density. The hyphae were straight and regular in shape. (b) (SEM × 4000) The majority of the hyphae was straight and smooth and exhibited intact surface without wrinkles. The microconidia were visible (red arrow). (c) (SEM × 8000). The hyphae were full and smooth with visible branches (red arrow). (d) (SEM × 1000) The hyphae density was smaller than that of the control group and became disorganized. (e and f) (SEM × 4000 and 8000) The majority of the hyphae were curved and partially expanded, and their surface was rough, shriveled, and wrinkled, with different sizes of depressions in some cases (black arrow).

With regard to SEM, the hyphae of *T. rubrum* without laser irradiation were dense and regular in shape, as shown in Figure 2(a) (×1000).  Figure 2(b) (×4000) indicates that the microconidia were visible and that the hyphae (red arrow) were straight and full with a smooth surface in the absence of wrinkles. The branching of specific hyphae (red arrow) was visible in Figure 2(c) (×8000). In comparison, the density of mycelia in the laser group was smaller than that of the control group and the hyphae were curved and disorganized (Figure 2(d), ×1000). Figures 2(e) and 2(f) (×4000 and ×8000) indicate that the surface of hyphae was no longer smooth and was characterized by atrophic regions (black arrow).

**Figure 3:**
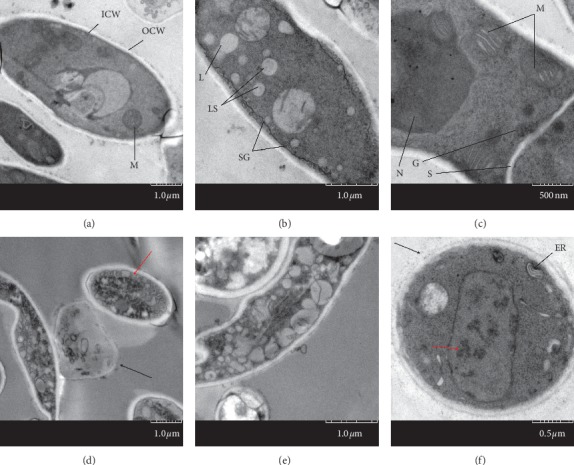
TEM observation of *T. rubrum* in the control and laser groups. (a) TEM (TEM × 1000) image of the longitudinal section of *T. rubrum* hyphae indicated double-layer cell walls and the outer cell wall (OCW) and the inner cell wall (ICW); organelles were noted in the cytoplasm. (b) (TEM × 5000) The cytoplasm was homogeneous with visible organelles, such as lysosomes (LS), liposomes (L), and some tiny secretory granules (SG). (c) (TEM × 8000) The mitochondria (M), nuclei (N), septum (S), and glycosome (G) could be observed clearly. (d–f) (TEM × 3000, 5000, and 8000) The structure of the double-layer cell wall was incomplete ((d), black arrow) and the cell membrane was discontinuous ((f), black arrow). The cytoplasm density was uneven ((d), red arrow). Although organelles, such as ER, were visible in the cytoplasm, other organelles were dissolved and the medullary bodies and protein coagulators were visible (manifestations of apoptosis, (f), red arrow).

TEM analysis demonstrated that, in the control group, the two membrane layers of the *T. rubrum* cell wall and the cell organelle were intact, the density of the cytoplasm was uniform, and the organelles, such as mitochondria, nucleus, septum, and glycosome, were visible (Figures 3(a)–3(c), ×3000, 5000, and 8000). In contrast to this pattern, the *T. rubrum* following laser irradiation of the two layers of the membrane structure was discontinuous and myeloid body and protein aggregates were formed, indicating the induction of apoptosis (Figures 3(d)–3(f), ×3000, 5000, and 8000). An abundance of vacuole and lipid droplets as well as blurred organelles were present in the uneven cytoplasmic density; however, its mycelial cells had intact wall (Figure 3(e), ×5000).

### 3.3. Transcriptome Analysis

RNA sequencing (RNA-seq) was used to compare the transcriptome differences between the laser irradiation and the control groups. Following removal of low-quality and trimming adapter sequences, 6.75–9.22 Gb 135-bp pair-end clean reads were generated from each library. Approximately 90% of the reads were mapped to the genome.

**Figure 4:**
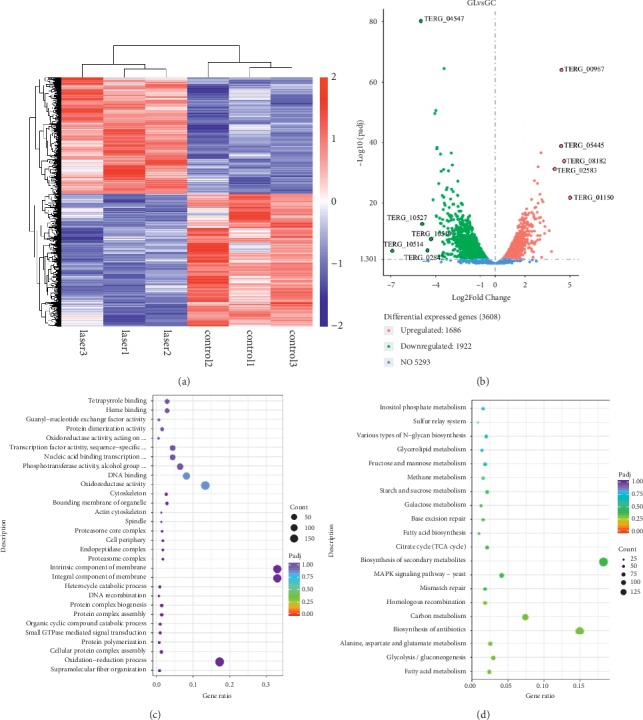
Differentially expressed genes between the laser and the control groups. (a) Heatmap of differentially expressed genes (DEGs) between the two groups. The 3 samples with laser treatment were clustered together, while the 3 samples in the control group were clustered into another group. (b) Volcano plot of DEGs between the two groups. The expression levels of Acyl-CoA N-acyltransferase (IPR016181, TERG_08182) and S-adenosyl-L-methionine-dependent methyltransferase (IPR029063, TERG_01150) were elevated in the laser irradiation group compared with those of the control group. (c) The DEGs were enriched in the GO terms associated with damage repair. The size of the circles represents the enriched gene count in the pathway; the colors indicate adjusted *p* values. (d) The DEGs were enriched in the KEGG pathways. The legend is the same as (c).

Heatmap of differentially expressed genes (DEGs) is shown in Figure 4(a), which indicates the 3 samples with laser treatment clustered together, while the 3 samples of the control group were clustered into another group.

The volcano plot (Figure 4(b)) intuitively displays the up- and downregulated genes after laser irradiation. The five genes with most fold-change in upregulated and downregulated are highlighted with black circles. Among these genes, the expression levels of Acyl-CoA N-acyltransferase (IPR016181, TERG_08182 in Figure 4(b)) and S-adenosyl-L-methionine-dependent methyltransferase (IPR029063, TERG_01150 in Figure 4(b)) were increased in the laser irradiation group. In addition, we identified 346 DEGs with a |log_2_ (fold change)| > 2 and adjusted *p* value < 0.05 (using the Benjamini–Hochberg algorithm) (Supplementary Table 1).

To fully understand the functions of the differentially expressed genes (DEGs), GO enrichment analysis was performed to identify important ontological categories associated with the expression levels detected (biological process, cellular component, and molecular functions). In addition, statistical enrichment of DEGs in the KEGG pathway was also used to investigate the function of DEGs.

Based on the GO enrichment results, the genes associated with the “intrinsic component of membrane,” “integral component of membrane,” “oxidation-reduction process,” “cytoskeleton,” and “bounding membrane of organelle” were enriched (Figure 4(c)).

In KEGG functional enrichment, the enriched pathways of DEGs are “biosynthesis of secondary metabolites,” “biosynthesis of antibiotics,” “carbon metabolism,” “MAPK signaling pathway-yeast,” “glycolysis/gluconeogenesis” etc. (Figure 4(d)).

## 4. Discussion

To date, the application of laser or intense pulse light irradiation on mycosis and onychomycosis has been reported in several studies [7, 20, 21]. However, the underlying mechanism remains unknown. In view of clinical importance of *T. rubrum* and since little is known about its morphology, an ultrastructural study of *T. rubrum* was performed and was used as a negative control experiment in an attempt to explore the treatment mechanism of Nd:YAG 1064 nm laser, which had previously proved its effect to inhibit the growth of *T. rubrum* (Figure 1(a)). In our study, under SEM, the hyphae of *T. rubrum* without laser irradiation were regular, straight with smooth surface. Under TEM, complete cell wall, bilateral membrane structure, and uniform density cytoplasm as well as visible organelles such as mitochondria and septa all appear normal. The ultrastructure we observed was consistent with that of the previous report [22, 23]. In the present study, specific conditions were used to laser irradiation on the colonies, and the ultrastructure was observed on the third day. SEM and TEM analyses clearly indicated that the membrane structure of the fungi was impaired by laser irradiation. Under laser irradiation, the myeloid body and protein aggregates, which are significant markers of apoptosis, were observed in the cytoplasm. The cell wall of genus *Trichophyton* contains a large amount of melanin which is susceptible to laser irradiations at the wavelength of 1064 nm [24]. Therefore, long-pulsed 1064 nm Nd:YAG laser acting on the chromophore group can cause an increase in temperature, leading to fungal destruction [25]. Some report also showed that different fungal strains have different sensitivities to laser because of the chromophore group and the damage of the laser to the strains is energy-dependent [25].

To elucidate the mechanism of growth inhibition caused on *T. rubrum* by laser irradiation, the transcriptome difference between the laser-irradiated and the control groups was compared. The results indicated that the expression levels of Acyl-CoA N-acyltransferase (IPR016181, TERG_08182 in Figure 3(b)) and S-adenosyl-L-methionine-dependent methyltransferase (IPR029063, TERG_01150 in Figure 3(b)) were increased in the laser irradiation group.

It has been shown in the previous studies that the function of acyltransferase is to catalyze lipid synthesis [26, 27]. The overexpression of diacylglycerol acyltransferase in the oleaginous yeast *Yarrowia lipolytica* affected the body size and the lipid number and distribution. In addition, lysophospholipid acyltransferase is considered the enzyme responsible for membrane remodeling in *Candida albicans* [28].

Furthermore, the GO terms “intrinsic component of membrane,” “integral component of membrane,” “oxidation-reduction process,” “cytoskeleton,” and “bounding membrane of organelle” in DEGs further indicated that the intracellular membrane system was in the different state between the laser irradiation and the control groups.

## 5. Conclusions

From these results, it is reasonable to infer that the membranes of *T. rubrum* were damaged by laser irradiation, and that the elevated expression levels of acyltransferase were triggered by the initiation of the damage repair pathway in this species. As the damage repair pathway is activated, the colony of the laser group can gradually restore its growth ability with time (Figure 1(b)).

## Data Availability

The data used to support the findings of this study are available from the corresponding author upon reasonable request.

## Supplementary Materials

Supplementary Materials Supplementary Table 1: a total of 346 DEGs were identified with a criterion |log_2_ (fold change)| > 2 and adjusted *p* value < 0.05 (using the Benjamini–Hochberg algorithm).

## References

[1] Weitzman I., Summerbell R. C. (1995). The dermatophytes. *Clinical Microbiology Reviews*.

[2] Aly R. (1994). Ecology and epidemiology of dermatophyte infections. *Journal of the American Academy of Dermatology*.

[3] Peres N. T. d. A., Maranhao F. C. A., Rossi A., Martinez-Rossi N. M. (2010). Dermatophytes: host-pathogen interaction and antifungal resistance. *Anais Brasileiros de Dermatologia*.

[4] Kim H. J., Park H. J., Suh D. H. (2018). Clinical factors influencing outcomes of 1064 nm neodymium-doped yttrium aluminum garnet (Nd:YAG) laser treatment for onychomycosis. *Annals of Dermatolog*.

[5] Kimura U., Takeuchi K., Kinoshita A., Takamori K., Hiruma M., Suga Y. (2012). Treating onychomycoses of the toenail: clinical efficacy of the sub-millisecond 1064 nm Nd:YAG laser using a 5 mm spot diameter. *Journal of Drugs in Dermatology*.

[6] Wanitphakdeedecha R., Thanomkitti K., Bunyaratavej S., Manuskiatti W. (2016). Efficacy and safety of 1064 nm Nd:YAG laser in treatment of onychomycosis. *Journal of Dermatological Treatment*.

[7] Gupta A. K., Simpson F. C. (2012). Medical devices for the treatment of onychomycosis. *Dermatologic Therapy*.

[8] Bhatta A. K., Huang X., Keyal U., Zhao J. J. (2014). Laser treatment for onychomycosis: a review. *Mycoses*.

[9] Vural E., Winfield H. L., Shingleton A. W., Horn T. D., Shafirstein G. (2008). The effects of laser irradiation on *Trichophyton rubrum* growth. *Lasers in Medical Science*.

[10] Ghavam S. A., Aref S., Mohajerani E., Shidfar M. R., Moravvej H. (2015). Laser irradiation on growth of *Trichophyton rubrum*: an *in vitro* study. *Lasers in Medical Science*.

[11] Kim Y. R., Lee Y. W., Choe Y. B., Ahn K. J. (2015). Lack of antifungal effect of 1064 nm long pulse Nd:YAG laser on the growth of *Trichophyton rubrum*. *Lasers in Medical Science*.

[12] Bolger A. M., Lohse M., Usadel B. (2014). Trimmomatic: a flexible trimmer for Illumina sequence data. *Bioinformatics*.

[13] Kim D., Langmead B., Salzberg S. L. (2015). HISAT: a fast spliced aligner with low memory requirements. *Nature Methods*.

[14] Liao Y., Smyth G. K., Shi W. (2014). FeatureCounts: an efficient general purpose program for assigning sequence reads to genomic features. *Bioinformatics*.

[15] Pertea M., Pertea G. M., Antonescu C. M., Chang T. C., Mendell J. T., Salzberg S. L. (2015). StringTie enables improved reconstruction of a transcriptome from RNA-seq reads. *Nature Biotechnology*.

[16] Love M. I., Huber W., Anders S. (2014). Moderated estimation of fold change and dispersion for RNA-seq data with DESeq2. *Genome Biology*.

[17] Warnes G. R., Bolker B., Bonebakker L. (2016). Gplots: various R programming tools for plotting data. https://github.com/talgalili/gplots.

[18] Young M. D., Wakfield M. J., Smyth G. K., Oshlack A. (2010). Gene ontology analysis for RNA-seq: accounting for selection bias. *Genome Biology*.

[19] Mao X., Cai T., Olyarchuk J. G., Wei L. (2005). Automated genome annotation and pathway identification using the KEGG Orthology (KO) as a controlled vocabulary. *Bioinformatics*.

[20] Xu Z. L., Xu J., Zhuo F. L. (2012). Effects of laser irradiation on *Trichophyton rubrum* growth and ultrastructure. *Chinese Medical Journal*.

[21] Huang H., Tang H., Huang M. (2018). Determining the optimal parameters of 420 nm intense pulsed light on *Trichophyton rubrum* growth *in vitro*. *Lasers in Medical Science*.

[22] Mares D., Romagnoli C., Sacchetti G., Vicentini C. B., Bruni A. (1998). Morphological study of *Trichophyton rubrum*: ultrastructural findings after treatment with 4-amino-3-methyl-1-phenylpyrazolo-(3,4-c)isothiazole. *Medical Mycology*.

[23] Yue X., Li Q., Wang H. (2015). An ultrastructural study of *Trichophyton rubrum* induced onychomycosis. *BMC Infectious Diseases*.

[24] Gupta A. K., Ahmad I., Borst I., Summerbell R. C. (2000). Detection of xanthomegnin in epidermal materials infected with *Trichophyton rubrum*. *Journal of Investigative Dermatology*.

[25] Cao Y., Xu S., Kong W., Xu Y., Fang H. (2020). Clinical retrospective analysis of long-pulsed 1064 nm Nd:YAG laser in the treatment of onychomycosis and its effect on the ultrastructure of fungus pathogen. *Lasers in Medical Science*.

[26] Gajdos P., Ledesma-Amaro R., Nicaud J. M., Certik M., Rossignol T. (2016). Overexpression of diacylglycerol acyltransferase in *Yarrowia lipolytica* affects lipid body size, number and distribution. *FEMS Yeast Research*.

[27] Polburee P., Ohashi T., Tsai Y. Y. (2018). Molecular cloning and overexpression of DGA1, an acyl-CoA-dependent diacylglycerol acyltransferase, in the oleaginous yeast Rhodosporidiobolus fluvialis DMKU-RK253. *Microbiology*.

[28] Ayyash M., Algahmi A., Gillespie J., Oelkers P. (2014). Characterization of a lysophospholipid acyltransferase involved in membrane remodeling in *Candida albicans*. *Biochimica et Biophysica Acta (BBA)-Molecular and Cell Biology of Lipids*.

